# Optimization of Signal Peptide via Site-Directed Mutagenesis for Enhanced Secretion of Heterologous Proteins in *Lactococcus lactis*

**DOI:** 10.3390/ijms231710044

**Published:** 2022-09-02

**Authors:** Nur Aqlili Riana Alias, Adelene Ai-Lian Song, Noorjahan Banu Alitheen, Raha Abdul Rahim, Siti Sarah Othman, Lionel Lian Aun In

**Affiliations:** 1 Department of Cell and Molecular Biology, Faculty of Biotechnology and Biomolecular Sciences, Universiti Putra Malaysia, Serdang 43400, Malaysia; 2 Department of Microbiology, Faculty of Biotechnology and Biomolecular Sciences, Universiti Putra Malaysia, Serdang 43400, Malaysia; 3 Laboratory of Vaccine and Biomolecules, Institute of Bioscience, Universiti Putra Malaysia, Serdang 43400, Malaysia; 4 UPM-MAKNA Cancer Research Laboratory, Institute of Bioscience, Universiti Putra Malaysia, Serdang 43400, Malaysia; 5 National Institutes of Biotechnology Malaysia, Argo-Biotechnology Institute Malaysia Complex, Serdang 43400, Malaysia; 6 Department of Biotechnology, Faculty of Applied Sciences, UCSI University Kuala Lumpur, Cheras 56000, Malaysia

**Keywords:** signal peptide, SPK1, USP45, site-directed mutagenesis, secretion efficiency, heterologous protein production, *Staphylococcal* nuclease (NUC), *Lactococcus lactis*

## Abstract

Secretion efficiency of heterologous proteins in the Generally Regarded As Safe (GRAS) *Lactococcus lactis* is often reported to be insufficiently low due to limitations such as poor targeting and translocation by the signal peptide or degradation by the host proteases. In this study, the secretion efficiency in the host was enhanced through the utilization of a heterologous signal peptide (SP) SPK1 of *Pediococcus pentosaceus*. The SPK1 was subjected to site-directed mutations targeting its tripartite N-, H-, and C-domains, and the effect on secretion efficiency as compared to the wild-type SPK1 and native lactococcal USP45 was determined on a reporter nuclease (NUC) of *Staphylococcus aureus*. A Fluorescence Resonance Energy Transfer (FRET) analysis indicated that four out of eight SPK1 variants successfully enhanced the secretion of NUC, with the best mutant, SPKM19, showing elevated secretion efficiency up to 88% (or by 1.4-fold) and an improved secretion activity yield of 0.292 ± 0.122 U/mL (or by 1.7-fold) compared to the wild-type SPK1. Modifications of the SPK1 at the cleavage site C-domain region had successfully augmented the secretion efficiency. Meanwhile, mutations in the H-domain region had resulted in a detrimental effect on the NUC secretion. The development of heterologous SPs with better efficacy than the USP45 has been demonstrated in this study for enhanced secretion of heterologous production and mucosal delivery applications in the lactococcal host.

## 1. Introduction

Research on recombinant secretory protein has intensified in the last decade to meet the ever-growing need for high-yield, high-quality, and active recombinant proteins for newly isolated enzymes and various other economically and pharmaceutically valuable proteins. The secretory expression system used for the manufacturing of these heterologous proteins must meet specific criteria such as consistent product quality and cost-effectiveness. Secretion, which directs the translocation of recombinant proteins across the plasma membrane directly into culture media, offers fast and straightforward purification and permits direct excess to the targeted environment (Pohl and Harwood, 2010) [1]. It is thus becoming a better means for vaccine delivery and large-scale industrial applications (Song et al., 2017) [2].

*Lactococcus lactis* is one of the most favoured and important Gram-positive prokaryotic cell factories for heterologous protein production besides hosts such as *Bacillus subtilis*, or Gram-negative *Escherichia coli* counterparts, owing to its monolayer cell well, absence of inclusion bodies, and simple proteolytic system comprising of only one extracellular housekeeping protease, HtrA, and one extracellular native protein, USP45 (Le Loir et al., 2005) [3]. This lactic acid bacteria (LAB), traditionally used in the food industry, has also advanced as a vaccine delivery carrier owing to its Generally Regarded as Safe (GRAS) status, as well as its probiotics and immunomodulating properties (Wang et al., 2016) [4]. Numerous advancements for expression developed in *L. lactis* have allowed for the high production of proteins intracellularly. Nevertheless, the overproduction of intracellular proteins often imposes certain limitations such as protein aggregation and misfolding, in addition to the expensive downstream purification of the products (Baradaran et al., 2013) [5]. Hence, many studies have now shifted to secretion to overcome those bottlenecks.

As much as secretion is the preferred strategy for the high-yield production of heterologous proteins and vaccine delivery in *L. lactis* (Fernandez et al., 2009) [6], difficulties in obtaining optimal secretion efficiency remain a major challenge in many prokaryotic hosts, including *L. lactis* (Zhang, Zhong, and Liang 2010) [7]. The secretion efficiency (SE), which is defined as the proportion of secreted proteins in relation to the total protein produced, is most often reported to be insufficiently low in the lactococcal host. Several factors that cause the low secretion efficiency of secreted proteins include inefficient translocation and improper protein folding due to the host secretory machinery and protein degradation due to the host proteases. Those factors are highly affected by the nature of the signal peptide and mature protein as well as the host strain, respectively (Le Loir et al., 2005; Westers, Westers, and Quax, 2004) [3,8]. 

Signal peptide (SPs), which is an N-terminal signaling sequence located upstream of a mature protein (MP) moiety, often shares a conserved tripartite structure comprised of the positively charged N-terminal, the hydrophobic core of the H-domain, and the non-polar C-terminal containing the cleavage site for signal peptidases. Each of the structural domains is known to play a specific role in the signal peptide exporting function. Additionally, each signal peptide reportedly has varying efficiency across the different proteins it is fused to (Freudl, 2018) [9]. In line with this, various random or site-directed mutagenesis targeting the tripartite structure have been performed in *L. lactis* to improve the performance of the SP, such as that observed for the most successful lactococcal signal peptide, USP45. The optimization of the SP’s inherent limitations had improved secretion yield by 51% compared to that of the native USP45 (Ng and Sarkar, 2013) [10]; however, the SE can still be improved further. On the other hand, several newly developed endogenous SPs such as SP310 (Ravn et al., 2000) [11], SPExp4 (Morello et al., 2008) [12], and SPAL9 (Ravn et al., 2003) [13] were produced, yet they were unable to mount higher SE than the native USP45, even after the SP was being engineered, such as in that observed for SP310mut2 (Ravn et al., 2003) [13]. On the other hand, the use of heterologous SPs such as the SLPA of *Lactobacillus brevis* was reportedly able to improve SE in *L. lactis*; however, the total protein yield remained low when compared to the lactococcal SP (Zhang et al., 2010) [7]. Therefore, the development of new SPs for applications in *L. lactis* is still impeding. 

In view of this, recently, Baradaran et al. (2013) had isolated a novel heterologous signal peptide SPK1 of *Pediococcus pentosaceus* strain K1, which showed the ability to produce comparable, if not better, secretion efficiency than the lactococcal USP45. Nevertheless, in silico analyses by Baradaran et al. (2013) indicated that there is potential to further enhance this signal peptide’s properties for improved secretion efficiency in *L. lactis*. The SPK1 had been previously shown to mediate the comparable secretion yield of several heterologous proteins such as a reporter GFP protein (Baradaran et al., 2013) [5], β-cyclodextrin glucanotransferase (β-CGTase) (Subramaniam et al., 2013) [14], *Staphylococcal* nuclease (Koko et al., 2019) [15], and xylanase (Roslan et al., 2020) [16], evidencing the promising potential of the SP to be further studied for improved secretion efficiency in *L. lactis*.

**Figure 1:**
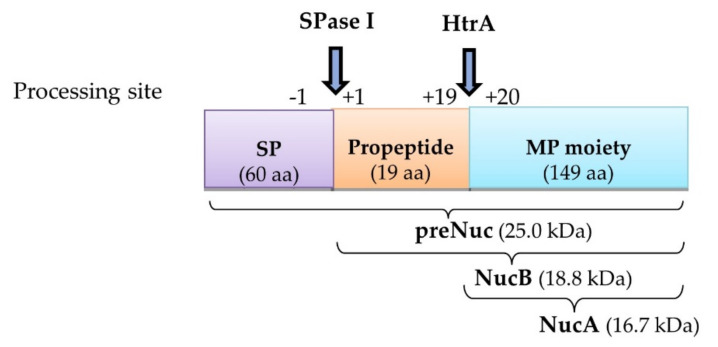
Schematic diagram showing different NUC forms produced following translocation. In *L. lactis*, the synthesized precursor NUC (preNuc), which consists of signal peptide (SP), native propeptide, and mature protein (MP) regions, matures into NucB through cleavage of the SP by SPase I (at processing site position −1). It was subsequently further processed into NucA through the removal of the 19-aa propeptide (at processing site position +19), postulated to be due to the activity of surface protease HtrA, before finally being secreted to the extracellular region.

Hence, in this study, the amino acid sequence of signal peptide SPK1 was optimized by targeting multiple site-directed mutagenesis (SDM) on the tripartite N-, H-, and C-domain regions, respectively. The effect of the SP SDM was determined on a prominent reporter model for secretion studies, the nuclease of *Staphylococcus aureus* (NUC), for which a rapid and sensitive in situ plate assay is widely available for screening of the enzymatic activity (Hu et al., 2013) [17]. The precursor enzyme of *Staphylococcal* NUC consists of a native signal peptide, a propeptide, and a mature protein, which has been previously reported to be efficiently expressed in different hosts, including *L. lactis*, where it would mature into two processed forms, NucB and NucA, following secretion due to the action of the host Sec machinery (Figure 1) (Le Loir et al., 1998) [18]. At the end of this study, the development of several novel derivatives of SPs with improved secretion efficiency than the USP45 were demonstrated for downstream applications in *L. lactis*. Additionally, an improved understanding of the role of the nature of signal peptide (SP) and mature protein (MP) moieties on protein secretion is also discussed.

## 2. Results

### 2.1. In Silico Analysis of SPK1 and Its Variants

**Figure 2:**
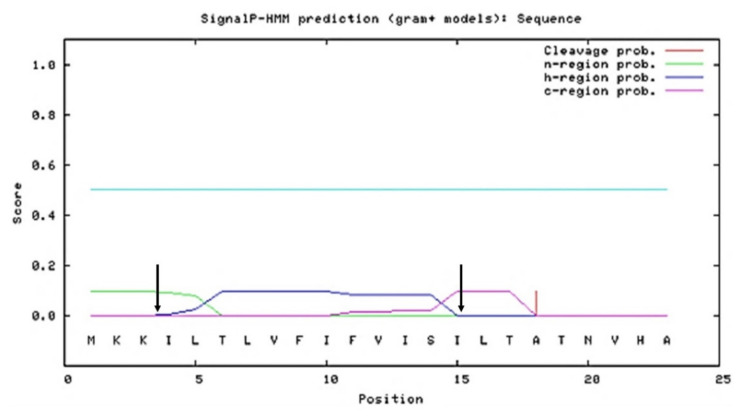
Graphical output of predicted N-, H-, and C-domain regions of the SPK1 using SignalP 3.0 server showing the SP harbour N-domain region from residue 1–3 (green line), H-domain region from residue 4–15 (blue line), and C-domain region from residue 16–23 (purple line). Arrow indicates separations between N-, H-, and C-domains, respectively.

In order to perform the desired site-directed mutations on SPK1, in silico characterization on the SP was initially performed. As shown in the SignalP 3.0 analysis (Figure 2), the amino acid sequence of SPK1 was found to harbour two polar basic residues, Lys (K) at positions 2 and 3 of the N-domain, which contributed to the overall net positive (+2) charge of the SP. Meanwhile, a stretch of non-polar hydrophobic residues with two polar uncharged residues, Thr (T) and Ser (S), were found at positions 6 and 14 of the H-core domain, and a cleavage motif sequence of Val-His-Ala (V-H-A) at the C-terminal domain was observed. Specific alterations on the N-, H-, and C-domains of SPK1 were performed. A total of 35 putative SPK1 variants containing mutations in either the N-, H-, or C-domain, as well as a combination of mutations in all three domains, was initially developed and subjected to screening by in silico analysis (Supplementary Table S1). Mutations were targeted on the signal peptide to increase the net positive charge at the N-domain, to increase hydrophobicity or incorporate helix-breaking residue such as Gly (G) at the H-domain, and to elongate or alter the cleavage site at the C-domain region.

As shown in Table 1, based on the signal peptide discrimination value, D-score, using SignalP4.0, of eight potential SPK1 variants, namely two H-domain SPK1 mutants (SPKM6 and SPKM20), five SPK1 variants harbouring C-domain mutations (SPKM9, SPKM16, SPKM17, SPKM19, and SPKM22), and one with N- and C-domain mutations (SPKM30) were successfully selected based on their higher D-score compared to the wild-type SPK1 (D > 0.781) with the exception of SPKM20, which had a lower D-score (D = 0.673). SPKM20 was still selected since the specific mutations made on the H-domain of the SP were previously reported to be able to augment protein secretion (Jonet et al., 2012) while other studies have also reported otherwise (von Heijne, 1990). All the SPK1 variants produced a higher D-score than the minimal threshold (D-score = 0.450), and Gram-positive SPs (Petersen et al., 2011) were recognized as functional SPs. Additionally, the mature moieties of all mutants were successfully predicted and recognized as secretory proteins. Based on the D-score value (Table 1), all putative SPK1 variants, except for SPKM20, were expected to perform better than the control USP45 in aiding NUC secretion, with those of the C-domain mutants being predicted to exhibit better performance than the H-domain and N-domain mutants, accordingly.

On the other hand, based on the physicochemical analysis (Table 1), the wild-type SPK1 was shown to harbour a pI value of 10.0 with an overall net positive charge balance (+2) contributed by the two Lysine (K) residues at the N-terminal. Compared to the control USP45, the SPK1 had higher hydrophobicity, aliphatic, and thermostability properties. This analysis was consistent with that previously reported by (Baradaran et al., 2013) [5], although there were slight variations in the values observed, believed to be due to the differences in the computational analysis tools used. Additionally, all the SPK1 variants showed lower hydrophobicity, aliphatic, and thermostability than the original SPK1 and none of the SPK1 variants showed a lower instability score than 40, indicating that all SPs are generally stable. Compared to the SPK1, all SPK1 variants retained the overall net positive charge (+2) following the introduction of specific mutations, except SPKM30, which had an increased net positive from charge +2 to +5. The SPK1 variants also retained a similar net negative charge (−1) at the first ten amino acids of the corresponding mature protein (MP) as the original SPK1. The resulting net charges on the SP and the MP moieties are in agreement with the ideal charge balance of an SP-MP that is usually observed for prokaryotes (Choo and Ranganathan, 2008) [19]. Following the modifications, four of the variants, SPKM17, SPKM19, SPKM22, and SPKM30, had an altered, new processing site of the Ala-X-Ala sequence at position −3 to −1 and an Ala (or Gly for SPKM17 only) at position +1. The effect of those SP modifications on NUC secretion was subsequently determined.

### 2.2. Recombinant NUC Were Secreted as Enzymatically Active Products in All Recombinants

**Figure 3:**
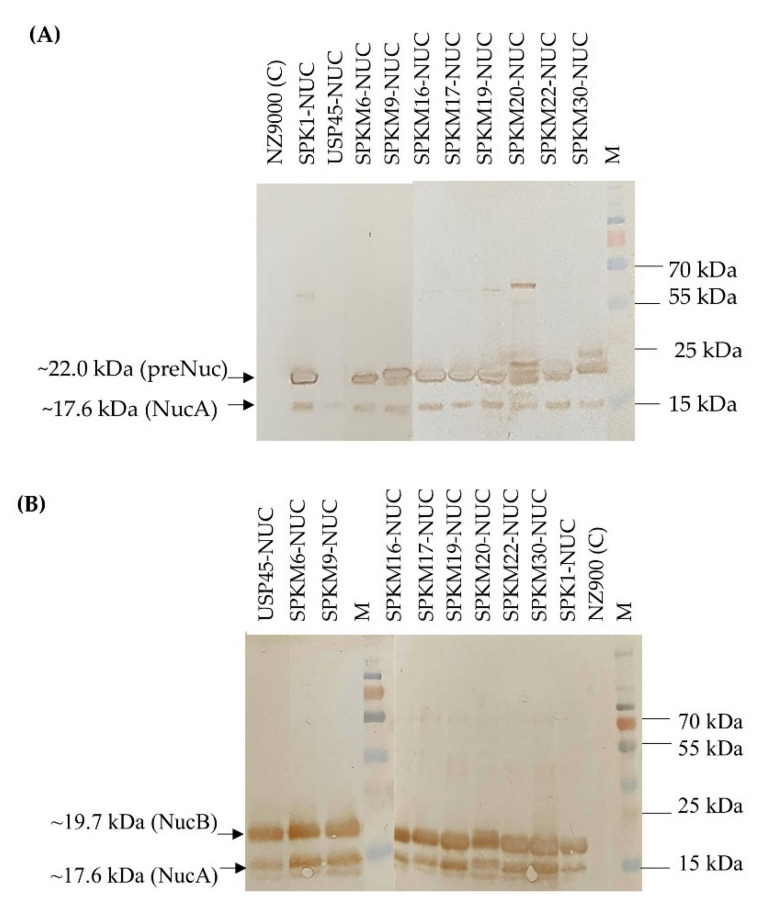
Western blotting analysis showing (**A**) intracellular fraction and (**B**) extracellular fraction of recombinant NUC protein produced by recombinants *L. lactis* harbouring different signal peptide constructs at 4 h expression with 40 ng/mL of nisin induction (unedited western blot is shown in the supplementary data, Scheme S1).

Ten constructs of recombinant *L. lactis* harbouring different signal peptides, namely, *SPK1*, *USP45*, and eight SPK1 variants (*SPKM6*, *SPKM9*, *SPKM20*, *SPKM16*, *SPKM17*, *SPKM19*, *SPKM22* and *SPKM30*) fused to the reporter *NUC(-NSP)* gene, which is devoid of its native signal peptide, were developed. As shown in Figure 3, following 4 h expression with 40 ng/mL nisin, all the recombinants *L. lactis* harbouring the different SP-NUC fusions had successfully expressed and secreted the target protein as indicated by the corresponding bands of the precursor NUC (preNuc) (~22 kDa) in the intracellular fraction and secreted NUC in the culture supernatant (the expected sizes of precursors for each recombinant is shown in Supplementary Table S2). The secreted NUCs were produced in two forms: a major NucB (19.7 kDa) and a minor NucA (17.6 kDa). NucB is the mature form of precursor Nuc, in which the SP has been cleaved off by SPase I following translocation (Figure 3). Subsequently, in *L. lactis*, NucB is further processed into NucA through the removal of the 19-aa native propeptide of the NUC mature moiety by housekeeping HtrA before being secreted to the extracellular environment. Both the secreted NUC forms, which are also absent in the negative control sample, have also been reportedly found in other studies (Le Loir et al., 1998; Poquet et al., 2000) [18,20]. The detection of the secreted NUC forms in all recombinants, which were absent in the negative control NZ9000 carrying empty pNZ8048, thus confirms that all the eight signal peptides’ SPK1 variants were functional and able to translocate the NUC protein to the extracellular region.

**Figure 4:**
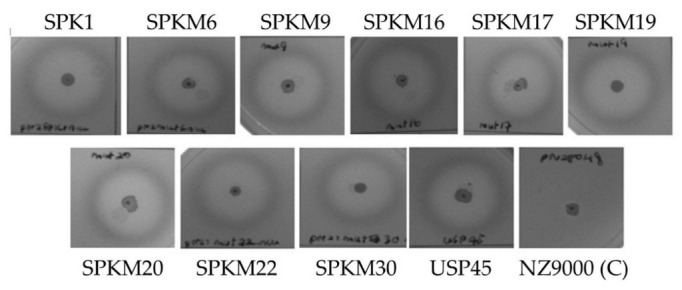
Nuclease activity plate assay showing halo zones’ formation of secreted NUC surrounding the colonies of recombinants *L. lactis*. The NZ9000 with empty plasmid pNZ8048 was used as a negative control.

Meanwhile, as shown in Figure 4, the secreted recombinant NUCs were enzymatically active, as indicated by the formation of halo zones surrounding colonies on the overlay BHI-TBD agar plate. As expected, the halo zone was absent in the control NZ9000-harboring, non-recombinant pNZ8048. NUC is a well-known thermostable deoxyribonuclease that could depolymerize single- or double-stranded DNA. The ability of the secreted NUC to depolymerize the DNA substrate in the media through the formation of the halo zone proves that the secreted target proteins of interest were indeed correctly folded and enzymatically active following secretion to the extracellular media.

### 2.3. Analysis of Secretion Efficiency and Yield of NUC by SPK1 Variants Showed Improved Secretion

**Figure 5:**
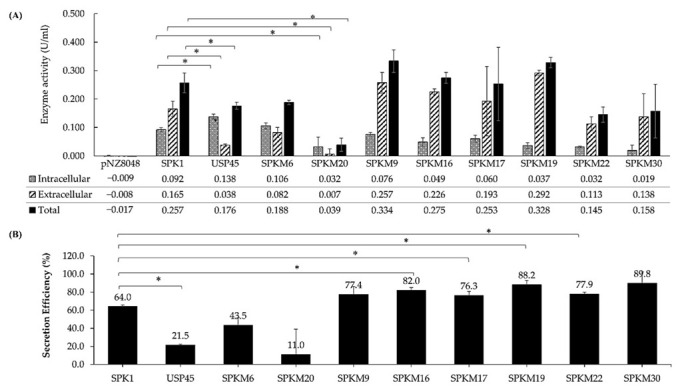
Fluorescence Resonance Energy Transfer (FRET) analysis of (**A**) intracellular, extracellular, and total activity, U/mL, of NUC enzyme by all recombinants. The detection of the intracellular enzyme activity was determined from the concentrated cell lysate, while the extracellular enzyme activity was determined directly from the filtered culture supernatant. The final activity of both fractions was reported after normalization to the dilution factors for accurate comparison. FRET analysis for (**B**) secretion efficiency, which is calculated as the percentage, %, of extracellular activity per total activity of NUC enzyme by all recombinants. The significance difference, *p* < 0.05, was shown as (*) for all groups when compared to the wild-type SPK1.

The effect of SDM on different domains (N-, H- or C-domains) of SPK1 was ultimately determined through quantification of the secretion yield and secretion efficiency of NUC activity using the FRET assay, where the one-unit activity of the fluorescence signal is corresponding with the one-unit activity of the DNA substrate produced from the NUC cleavage at 37 °C (Kiedrowski et al., 2011) [21]. In this study, the SPK1 was mutated at its H-core domain by increasing the net hydrophobicity (SPKM6) or by the addition of the helix-breaking residues Gly (G) at the middle core domain (SPKM20). Based on the enzyme activity analysis (Figure 5), both of the H-domain mutants were unable to enhance the secretion efficiency of NUC in comparison with SPK1. Both the SPKM6 and SPKM20 produced SE (which refers to the proportion of secreted proteins per total proteins) ratings of 43% and 11%, respectively (Figure 5B), which indicates inefficient secretion of the NUC enzyme with a majority of the proteins retained in the cytoplasm instead of being secreted out to the culture supernatant. For SPKM20, a significantly lower yield of intracellular and total NUC activity compared to SPK1 was also observed (*p* < 0.05), indicating a detrimental effect on both precursors and secreted protein productions.

Additionally, the SPK1 was also mutated at its C-terminal domain through either an increase in the length of the domain region (SPKM9, SPKM16) or the altering of the cleavage site of the SPase I region at position −3 to +1 (SPKM17, SPKM19, and SPKM22). Based on Figure 5, all the C-domain mutants had successfully produced non-significant (*p* > 0.05) higher extracellular NUC activity than the intracellular ones when compared to SPK1. Four of the C-domain mutants carrying altered SPase I cleavage sites (SPKM16, SPKM17, SPKM19) and a longer C-domain region (SPKM22) showed significantly improved SE by approximately 1.1 to 1.4-fold than the SPK1 (*p* < 0.05). Of those with increased SE, SPKM19 showed the highest increment with an elevated SE of up to 88% (or by about 1.4-fold) and an improved secreted activity yield of 0.292 ± 0.122 U/mL (or by about 1.7-fold) compared to the native SPK1. The ability of those C-domain mutants to exhibit more enhanced SE than SPK1 is consistent with the previous in silico analysis, which also showed higher D-score values by all those mutants compared to SPK1 and other domain mutants in this study. Based on this finding, it was shown that mutations on the C-domain had a positive effect in augmenting NUC secretion in *L. lactis*, at least on the SE. Since SPKM19 showed a better capability in aiding the secretion of NUC than the other C-domain counterparts, it was thereby selected as the best SPK1 variant for further analysis in this study.

The synergistic effect of the combined N- domain and C-domain mutation through an increment in the net positive charge by insertion of polar residues (KKK) and the alteration of the SPase I cleavage site at the C-domain, respectively, was also performed, yielding SPKM30. While an increment in the SE was observed for SPKM30, it was not statistically significant compared to the SPK1 (*p >* 0.05). Lastly, analysis of the USP45 control (Figure 5B) showed that, although it is the most commonly used signal peptide in *L. lactis*, poor SE was shown with SE ~21.5%, or as 3-fold lower than that of SPK1 (*p* < 0.05). The yield of secreted NUC activity for USP45 was also about four-fold lower (0.038 ± 0.004 U/mL), and total NUC activity was lower by about 1.4-fold lower (0.257 ± 0.035 U/mL) than that for SPK1 (*p* < 0.05). A high percentage of NUC (~79%) was found to be in the intracellular fraction for USP45, making it the only SP other than SPKM6 and SPKM20 which had a higher fraction of intracellular compared to extracellular proteins. Meanwhile, when comparing USP45 to the SPK1 variants, all except SPKM20 showed superior performance in regard to secretion efficiency for NUC secretion. The findings supported the previous in silico analysis based on the D-score values, which reported the D-score of USP45 to be the lowest compared to SPK1 and other mutants except SPKM20 in this study.

## 3. Discussion

In this study, a preliminary in silico prediction of the efficiency of the newly designed putative signal peptides had been performed prior to experimental analysis to eliminate the chance of developing non-functional and low efficacy signal peptides (SPs). The characteristics and functionality of the SPK1 and its variants were analyzed using several reliable computational tools. SignalP 4.0 is an efficient SP prediction tool used in this study that works similar to SignalP 3.0 software but is superior in its ability to discriminate between secretory and non-secretory proteins by including the transmembrane (TM) region in the prediction (Petersen et al., 2011) [22]. SignalP 4.0 is also superior compared to 15 other software programs, including PrediSi, SPEPlip, Signal-BLAST, Philius, Phobius, and Spoctopus, and SignalP 3.0 in the SP prediction of all three organisms, namely, Gram-positive, Gram-negative, and eukaryotes (Peterson et al., 2011) [22]. Meanwhile, the SP tripartite region (N-, H-, and C-domain) was analyzed by SignalP 3.0, a feature which was lacking in SignalP 4.0. Several output scores were generated following the deposition of an amino acid sequence of potential secretory proteins with one D-score value, which is critical for the selection of the SPK1 variant in this study.

The D-score is implemented as the weighted average of the S-mean (calculated for actual signal peptide) and Y-max score (calculated for cleavage site). A D-score above the set minimum threshold indicates the positive prediction of a signal peptide, and it subsequently discriminates a secretory from a non-secretory protein. A higher D-score value is also associated with the better ability of the fusion SP-protein to be recognized as a secretory protein through recognition by SPase I at the later stage of the translocation process. The ideal cut-off of the D-score to determine the input sequence containing an SP in Gram-positive prokaryotes is 0.45 for the SignalP-TM (included transmembrane) and 0.57 for the SignalP-noTM (no transmembrane) networks (Nielsen, 2017) [23]. In this study, the computational analysis performed on all eight designed SPK1 variants showed that they are each functional as an SP and are able to direct more efficient secretion of NUC than SPK1 and USP45.

Ideally, in Gram-positive bacteria, the signal peptides would have a median net charge of +3, an aliphatic index between 75 and 200, an isoelectric point (pI) with median values of 10.3, and a GRAVY score of 93.5% from hydropathicity calculations or lean towards hydrophobic propensity. Meanwhile, the mature proteins (MPs) of Gram-positive bacteria are characterized to have a median net negative charge, aliphatic index between 50 and 100, and a GRAVY score of 94.6% or lean towards hydrophilic propensity (Choo and Ranganathan, 2008) [19]. The net positive charge of SP is important in prokaryotes for effective interaction with the negatively charged phospholipid bilayer during the initial step of translocation across Sec translocon (Peng et al., 2019) [24]. The net positive charge balance on the SP is required to be higher than the global net positive charge of the MP to ensure the correct orientation of the SP-MP during the late translocation step (von Heijne, 1990; von Heijne, 1986) [25,26]. It is presumed that the positively charged SP remains embedded within the plasma membrane while the more negatively charged MP protrudes towards the extracytoplasmic fraction during the final translocation step for subsequent processing by chaperons such as SPase I (Choo and Ranganathan, 2008) [19]. 

In this study, site-directed mutations on SP had been performed to understand the role that each tripartite domain plays in protein secretion. The effects of the SP on secretion efficiency were tested on a 23 kDa-nuclease of *Staphylococcus aureus* as a reporter protein. NUC is a genetically and biochemically well-characterized endonuclease that is commonly used in secretion studies as it is intrinsically thermostable and non-toxic, and sensitive plate assays are readily available for the rapid detection of the enzyme activity in situ (Cuatrecasas, Ftjchs, and Anfinsen 1967) [27]. Under the influence of the promoter nisin and the host Sec-machinery, the expression and secretion of the NUC as an active and correctly folded enzyme have been successfully demonstrated by all *L. lactis* recombinants. In this study, the two intracellular NUC forms (preNuc and NucA) were found in the intracellular fraction, where only NucA was previously reported to be enzymatically active (Le Loir et al., 1998) [18]. Meanwhile, both secreted NUC forms (NucB and NucA) that were found in the extracellular fractions have been previously reported to be enzymatically active (Le Loir et al., 1998) [18]. Thus, the enzymatic activity detected from the FRET assay was thus presumed to be the activity of NucA for intracellular activity and both NucA and NucB for extracellular activity, respectively.

FRET is a rapid and highly sensitive assay that permits the detection of a small number of proteins in the culture supernatant. The transmission of energy from the donor molecule to the acceptor molecule served as the underlying principle for fluorescence signal detection. The fluorescently labelled DNA probe that served as a substrate for NUC in this study has been modified to consist of a 15-basepair oligonucleotide tagged with a Cy3 fluorophore (donor molecule) at the 3′-end and a Black Hole Quencher 2 (BHQ2) (acceptor molecule) at the 5′-end (Kiedrowski et al., 2011) [21]. In the absence of the NUC enzyme, the BHQ2 quencher molecule retains proximity with the Cy3 fluorophore, constitutively quenching the signals emitted by the Cy3 molecule and thus resulting in no signal detection. Subsequently, in the presence of the NUC enzyme, it digests the middle of the DNA strand, breaking the proximity between the BHQ2 quencher and Cy3 fluorophore thus permitting the emission of fluorescent signals, which can be detected at 585 nm. The FRET assay used in this study ensures that only enzymatically active proteins of NUC were detected.

An understanding of the nature of the SP is essential for the development of an enhanced secretion system, as each tripartite domain structure of SP serves a certain role in the SP function. The C-domain of an SP has an essential role in the last stage of the translocation process by interacting with the signal peptidases (SPases) through its cleavage recognition site for SP cleavage and subsequent release of the protein mature moiety to the extracellular environment. In prokaryotes, all secretory proteins except lipoproteins are processed by signal peptidase type I (SPase I). Studies have shown that there are specific requirements for the SPases’ activity at the cleavage site position. While the H-domain of the SP usually adopts an α-conformation, the C-domain requires a β-stranded conformation for recognition by the SPases (van Roosmalen et al., 2004) [28]. Additionally, there is also high selectivity for small and aliphatic residues at positions −3 and −1 of the cleavage site, with Ala (A) predominating those regions in the SPs of Gram-positive and Gram-negative prokaryotes (Choo and Ranganathan, 2008) [19], which is postulated to be the preferred recognition site for the SPase I. This so-called “−3, −1 rule, A-X-A rule” has been first postulated by von Heijne (1986) [26]. In this study, the alteration of the SP C-domain region via the cleavage site was shown to successfully enhance the secretion efficiency in *L. lactis.*

Site-directed mutation through the insertion of the Ala residue at the −3 position of the SP cleavage site to adopt the consensus motif A-X-A (−3, −1), as shown for SPKM17, had successfully elevated NUC secretion in regard to secretion efficiency. More interestingly, the addition of Ala at an additional position, +1, of the mature protein to adopt the A-X-A-A sequence motif (here proposed as the −3, −1, and +1 rule), as shown for SPKM19, had given a more prominent effect in enhancing secretion with SE, with a value of 88% for NUC secretion. Choo and Ranganathan (2008) [6] have previously demonstrated that the SPase I recognition site that begins at the −3 position extends up to the +1 position of the cleavage site region. Thus, in this study, the sequence motif, Ala-X-Ala-Ala at positions −3, −1, and +1 of the cleavage site is postulated to serve as a better recognition site for the SPase I in *L. lactis*. It is presumed that the higher cleavage efficiency of SPs is proportional to a higher release of mature protein to the extracellular region following translocation. Of note, all C-domain recombinant mutants including SPKM19 retained the overall net positive charge (+2) of the SP and net negative charge (−1) of the first 10 residues of the MP moieties.

Other C-domain mutations such as SPKM16 and SPKM22 also consistently showed higher secretion efficiency than the wild-type SPK1. SPKM22 harboured a similar motif sequence, A-X-A-A (−3,−1,−1,+1 position), at the C-domain, similar to SPKM19, but with the additional insertion of a Pro at position −6, and showed enhanced SE of nearly 80% of NUC. It was also previously suggested that there is a requirement for a helix-breaking residue such as Pro (P) at the C-domain of an SP (van Roosmalen et al., 2004) [28]. This helix-breaker served as a structure disrupter due to its steric hindrance from a cyclic side-chain and inability to form hydrogen bonds, forming a turn at the end of the C-domain to nucleate β-conformation, which is ideal at the C-domain for SPase I recognition. van Roosmalen et al. (2004) and Tjalsma et al. (2004) [28,29] have previously suggested a need for turn-promoting residues such as Gly (G) and Pro (P), especially at position −6 to −4 relative to the SP cleavage site and at position +2 of the mature protein to promote a short β-conformation. Similarly, Auclair, Bhanu, and Kendall (2012) [30] reported that the formation of a short β-conformation at positions −5 and −1 relative to the SP cleavage site has served as an active binding site for SPase I. Based on the finding, the C-domain of the signal peptide via its cleavage site has a critical role in enhancing secretion efficiency in *L. lactis*.

In this study, mutation at the H-domain regions, through the incorporation of helix-breaking Gly (SPKM6) or by increasing the hydrophobicity of the domain (SPKM20), was unable to improve secretion efficiency in *L. lactis*. The poor SE of NUC shown by both of the mutants was in agreement with the initial in silico analysis (D-score). Compared to other SPs, SPKM20 had the lowest D-score, while SPKM6 is only higher than the USP45 and is the same as the wild-type SPK1. The H-domain of an SP has an important role during translocation by interacting with the hydrophobic plasma membrane (von Heijne, 1990) [25]. The H-domain of an SP usually adopts an α-helical conformation in the membrane due to a stretch of hydrophobic residues. Several mutagenesis studies, by increasing the hydrophobicity or extending the length of an SP H-domain, have shown an increased rate of export (Chou and Kendall, 1990) [31]. In both *E. coli* and *Bacillus brevis*, increasing hydrophobicity at the H-domain with the addition of Leu (L) or Phe (F) residues improved the secretion of phoA and IL-2, respectively (Takimura et al., 1997) [32]. Additionally, the presence of a charged helix-breaking residue such as Gly (G) or Pro (P) is frequently found in the middle hydrophobic stretch of the SP H-domain of Gram-negative (found in about ~60% in *B. subtilis*) and is thought to promote the formation of a hairpin-like loop structure for insertion into the plasma membrane during translocation (Tjalsma et al., 2000) [33]. The hairpin loop model for the insertion of SP across a membrane was first postulated by von Heijne (1990) [25]. In contrast, here, for both of the mutant H-domain, a majority of the protein products were found to remain in the cytoplasm instead of being secreted out.

The negative effect of increasing the hydrophobicity of the H-domain on secretion, as shown in this study, is similar to a study by Ravn et al. (2003) [13], which also showed a decrease in SE in *L. lactis* following an increase in the hydrophobicity of the H-domain using the mutant SP SP310. This indicates that there is a species-specific requirement for the turn-motif residues in the SP H-domain of different prokaryotes. Meanwhile, the negative effect of the turn-motif Gly (G) at the H-domain, as shown in this study, is supported by a recent study by Mahmud et al., 2019 [34], which showed that the insertion of Gly (G) at the H-domain of SP M5 had resulted in a decrease in β-CGTase secretion in *L. lactis*, although they initially showed a positive effect on the SE of the enzyme in *E. coli* (Jonet et al., 2012) [35]. These discrepancies further suggest that the SP H-domain has a less critical role in affecting secretion efficiency, at least in *L. lactis*. In fact, in cases such as SPKM20 (SE, 11%), altering the H-domain may detrimentally affect protein secretion.

Aside from the role of an SP, the nature of a mature protein (MP) region is also essential in protein secretion. The development of new SPs is often challenged by the possibility of having an incompatible SP with the fusion protein. This is because, while the presence of a strong signal peptide is essential for protein secretion, it does not necessarily guarantee successful and efficient secretion. Varying efficiencies of the signal peptide across the different proteins used have been observed (Freudl, 2018) [9]. Recently, the first 10 residues and the first 18 residues of the MP, at least, have been shown to be equally critical in the translocation and secretion of proteins (Kajava et al., 2000; Choo and Ranganathan, 2008) [19,36]. In bacteria, both SP and MP moieties were shown to harbour a certain charge balance that surrounds the cleavage site, with a majority of SPs bearing a net positive charge, while a majority of MPs inclined towards a net negative charge (Choo and Ranganathan, 2008) [19]. This striking difference in the net charge balance between the SP and MP moieties is presumed to be conserved in secretory proteins to enable their interactions with other players in the secretion pathway. The important role of the first 10 MP net charge has also been demonstrated by another study through fusion with a propeptide LEISSTCDA. Le Loir et al., 2001 [37], showed that fusion of the anionic linker LEISSTCDA in between the SP and the MP moieties enables improved protein secretion, which is believed to be caused by an increase in net negative charge balance surrounding the cleavage site. The enhancement effect of the LEISSTCDA on protein secretion was said to be independent of the SP used.

## 4. Materials and Methods

### 4.1. In Silico Characterization and Design of Site-Directed Mutation of Signal Peptide

Signal peptide SPK1 was characterized by depositing the amino acid sequence of the signal peptide fused to NUC lacking its signal peptide as a target protein into SignalP 3.0 (Bendtsen et al., 2004) [38] http://www.cbs.dtu.dk/services/SignalP-3.0/ (accessed on 3 August 2019) to determine the SP tripartite N-, H-, and C-domain regions. The parameter was set against Gram-positive organisms using both neural networks and hidden Markov models. The appropriate mutations that include modifications on one region or a combination of two or more (N-, H-, or C-domain) regions within the SPK1 sequence were subsequently designed, targeting an increase in the N-domain positive charge, an increase or decrease in the H-domain hydrophobicity, and modifications on the C-domain cleavage site. The designed putative SPK1 variants were subjected to screening via the SignalP 4.0 server (Petersen et al., 2011) [22] http://www.cbs.dtu.dk/services/SignalP-4.1/ (accessed on 1 July 2019) to predict the signal peptide probability and function. Selected SPK1 variants with SP probability, a D-score, higher than the minimum threshold of WT SPK1 (D score > 0.75) were chosen and subjected to further in silico analysis. The SP physicochemical properties including molecular weight, theoretical pI, amino acid composition (number of negatively charged and positively charged residues), instability index, aliphatic index, and grand average of hydropathicity (GRAVY) index were performed using the ExPASy ProtParam server (Wilkins et al., 2005) [39] http://web.expasy.org/protparam/ (accessed on 1 July 2019) Besides that, using the deposited amino acid sequence of SP-NUC fusion proteins, the prediction of subcellular localization of secretory proteins (cell membrane, cell wall, cytoplasm, or extracellular) was determined using the pLoc_bal-mGpos server (Xiao et al., 2019) [40] http://www.jci-bioinfo.cn/pLoc_bal-mGpos/ (accessed on 12 November 2020), the types of recognizable signal peptidases (Sec/SPI, Sec/SPII or Tat/SPI) were determined using SignalP 5.0 server (Almagro et al., 2019) [41] http://www.cbs.dtu.dk/services/SignalP/ (accessed on 12 November 2020), and the secondary and tertiary structure analysis was performed using a RaptorX prediction server (http://raptorx.uchicago.edu/ContactMap/) (accessed on 12 November 2020). 

### 4.2. Bacterial Strains and Plasmids

All bacterial strains and plasmids are listed in Supplementary Table S3. The *L. lactis* NZ9000 used as an expression host was cultured in M17 media supplemented with 0.5% (*w*/*v*) glucose (GM17) and incubated at 30 °C as a standing culture for 16–18 h. Meanwhile, the *E. coli* TOP10 strain used as propagation host was grown in Luria-Bertani (LB) media and incubated at 37 °C with agitation at 250 rpm for 16–18 h. Selection of putative transformants harbouring the pNZ8048 expression plasmid and the pCR^TM^-Blunt II-TOPO^®^ cloning plasmid were performed with 7.5 µg/mL of chloramphenicol (Cm) and 50 µg/mL of kanamycin, respectively.

### 4.3. Synthesis and Amplification of Signal Peptides and Reporter Gene

The oligonucleotide sequences of signal peptides were obtained; the controls SPK1 (MKK ILT LVF IFV ISI LTA TN VHA) of *Pediococcus pentosaceus* strain K1 (Baradaran et al. (2013)) [5] and USP45 (MKK KII SAI LMS TVI LSA AAP LSG VYA) of *L. lactis* subsp. *cremoris* MG1363, and the other eight putative SPK1 variants developed in this study (SPKM6, SPKM9, SPKM16, SPKM17, SPKM19, SPKM20, SPKM22, and SPKM30), were chemically synthesized (Integrated DNA Technologies, Coralville, IA, USA). The gene coding sequence of the target protein, the NUC of *S. aureus* subsp. *aureus* strain N315 (GenBank Accession No: BAB41979.1), was synthesized in the lyophilized form of a commercialized plasmid harbouring the gene, i.e., pBSK(+): SimpleAmp-Saureus*Nuc* (Biomatik, Wilmington, DE, USA). The full length of the *Nuc* gene (684 bp) encodes for a 228 polypeptide (60 residues N-terminal native signal peptide, 19 residues native pro-peptide, and 149 residues mature protein region). All SPs and target proteins were PCR amplified using specific primers as listed in Supplementary Table S4. For SP, the primers were incorporated with *Nco*I and *Kpn*I RE sites at the N- and C-terminals, respectively. While for the target gene of interest, the *Nuc* gene, which is devoid of its native signal peptide, the primers were incorporated with *Kpn*I and *SacI* at the N- and C-terminals, respectively. Additionally, a six*-Histidine* (*6x-His*) tag sequence was included at the C-terminal end for the selection of the expressed protein. The PCR amplification was performed with a 20 µL reaction mixture containing 1.25 U *Pfu* polymerase (Thermo Fisher Scientific, Waltham, MA, USA), 1X *Pfu* Buffer with MgSO_4_, 0.25 µM of the forward primer and reverse primer, 0.25 mM dNTP Mix (Thermo Fisher Scientific, USA), ~0.1–50 ng/µL of the DNA template, and sterile distilled water.

### 4.4. Construction of SP-NUC Secretory Cassettes

**Figure 6:**
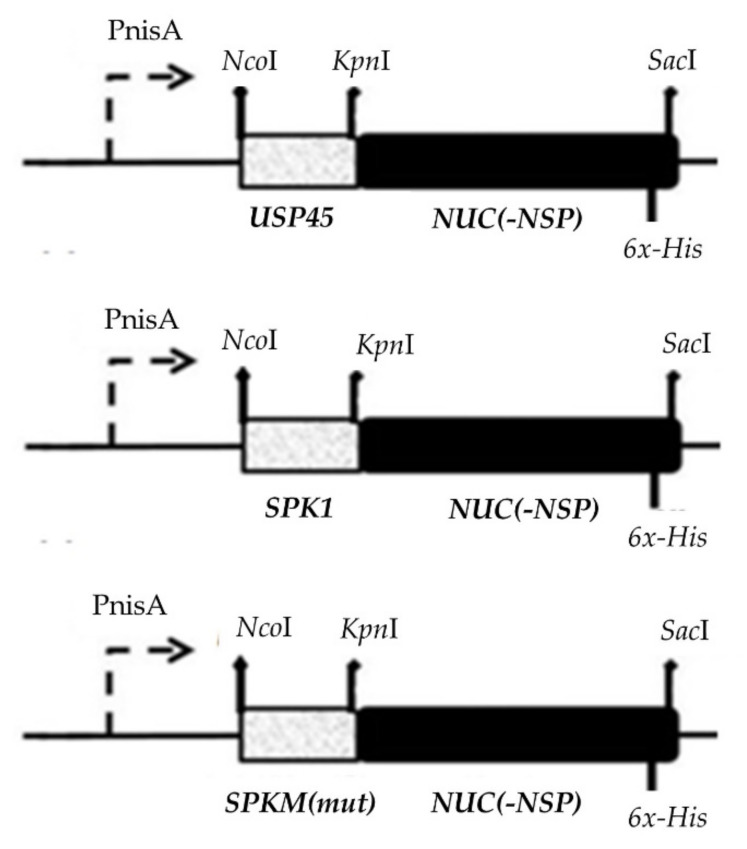
Construction of secretory plasmids harbouring different SP-NUC cassettes. The secretory cassettes of different control SPs (SPK1/USP45) and different SPK1 variants (termed SPKM) fused to the NUC gene were placed downstream of the nisin inducible promoter (P_nisA_) of pNZ8048. The respective REs (*Nco*I, *Sac*I, and *Kpn*I) used for cloning of the genes and a six-histidine (6x-His) tagged sequence included at the C-terminal end of the NUC gene upstream of a stop codon for detection of the target protein are shown.

The construction of *SP-NUC* secretory cassettes is shown in Figure 6. All purified PCR products of SPs were single digested with *Kpn*I RE and ligated to similarly digested reporter *Nuc.* Ligation of *SP-NUC* cassettes was carried out with a T4 DNA Ligase (Thermo Fisher Scientific, USA) at 25 °C for 4 h and ligation products were purified by gel electrophoresis. Successful ligations were determined by PCR amplification using designated forward primers specific to their respective SPs and the reverse primer of the *Nuc* gene and analyzed on agarose gel electrophoresis. In order to increase the chance of successful transformation in host *L. lactis* NZ9000, the *SP*-*NUC* secretory cassettes were initially cloned in *E. coli* Top10 cloning vectors (*pEASY*^®^-Blunt Zero or pCR^™^-Blunt II-TOPO^®^ Thermo) according to the manufacturer’s protocol for propagation before subcloning into the *L. lactis* NZ9000 expression plasmid, pNZ8048.

### 4.5. Cloning and Transformation into L. lactis Host

*L. lactis* NZ9000 was made electrocompetent according to the protocol described by Holo and Nes (1989) [42] with minor modifications. An overnight culture of *L. lactis* NZ9000 was diluted to 1:10 into fresh 40 mL SGM17 broth (GM17 containing 0.5 M sucrose) supplemented with 2% (*w*/*v*) glycine (Vivantis, Shah Alam, Selangor, Malaysia) and allowed to grow until it reached OD_600nm_ = ~0.5 (about 6–7 h). The cells were harvested by centrifugation at 4000× *g* at 4 °C for 10 min. The supernatant was decanted, while the cell pellet was gently resuspended in half volume of ice-cold 0.5 M sucrose supplemented with 10% (*v*/*v*) glycerol followed by centrifugation at 4000× *g* for 10 min at 4 °C to wash the pellet. The cell pellet was then treated with half volume (20 mL) of 0.5 M sucrose supplemented with 10% (*v*/*v*) glycerol and incubated on ice for 20 min before centrifugation at 4000× *g* for 10 min at 4 °C. The supernatant was decanted, whereas the cell pellet was resuspended in 1/10 culture volume of ice-cold 0.5 M sucrose supplemented with 10% (*v*/*v*) glycerol and aliquoted into volumes of 40 µL for use in transformation.

Transformation into a competent *L. lactis* NZ9000 host was conducted using the electroporation method as described by Holo and Nes (1989) [42] with some modifications. Volumes of ligation mixtures (4 μL of recombinant pNZ8048 and 2 μL of empty pNZ8048 as control) were mixed with 40 µL of thawed competent *L. lactis* NZ9000 cells. The reaction mixtures were transferred into an ice-cold sterile 0.2 cm-gap electroporation cuvette (Bio-Rad Laboratories, Hercules, CA, USA) and incubated on ice for 20 min before being electroporated at 2.0 kV, 25 µF capacitance, and 200 Ω resistance with ~4.5–5.0-ms^−1^ time constant using Gene Pulser Xcell™ (Bio-Rad Laboratories, USA). The volume of 0.96 mL of ice-cold SGM17 broth supplemented with 20 mM MgCl_2_ and 2 mM CaCl_2_ was added to the cuvette immediately after pulsing and placed on ice briefly before being incubated at 30 °C as a standing culture for 2 h for cell recovery. Sample mixtures were grown on GM17 agar supplemented with 0.5 M sucrose and 5 µg/mL Cm at 30 °C for 2–3 days for the selection of the putative transformants. Putative transformants were confirmed by sequencing the inserts.

### 4.6. Expression and Secretion Condition of Recombinants L. lactis

Protein expression was performed using overnight cultures of recombinant *L. lactis* diluted (1:10) in 40 mL of fresh M17 broth supplemented with 0.5% glucose (GM17) and 7.5 µg/mL Cm. The culture was grown until OD_600nm_ = ~0.5 (mid-log phase) before being added with 40 ng/mL nisin to induce expression. The culture was incubated for another 4 h before centrifuging at 4000× *g* for 10 min at 4 °C to harvest the cells (optimization of the culture condition was initially performed as shown in the Supplementary data, Scheme S2). Intracellular proteins were extracted from cell pellets by washing twice in a 1/10 culture volume of ice-cold sterile distilled water and ice-cold 1X PBS, respectively. For every washing step, the cell suspensions were centrifuged at 4000× *g*, 10 min at 4 °C, and the supernatants were carefully decanted. The cell pellet was recovered in a 1/10 culture volume of ice-cold 1X PBS. Volumes of 1 mL cell suspension proceeded to sonication with an Omni Ruptor 4000 Ultrasonic Homogenizer (Omni International, Tulsa, OK, USA) using a 3 mm diameter probe. Cells were kept on ice all the time and were homogenized at 40% power and pulsed for 8 min with 30 s delay for a total of 3 cycles until the lysate became clear. Samples were centrifuged at 4000× *g* at 4 °C for 10 min, and the supernatant containing crude intracellular proteins was stored until further analysis.

Meanwhile, extraction of extracellular proteins from culture supernatant was conducted according to Koontz (2014) [43] with minor modifications. The culture supernatant was precipitated in 1/10 of culture volume with 100% (*w*/*v*) TCA (Merck, Darmstadt, Germany), briefly vortexed and incubated on ice for 2 h, before being centrifuged at 14,000× *g* for 10 min at 4 °C. The supernatant was discarded while the extracellular proteins’ pellet was washed twice in 1/10 culture volume of 100% (*v*/*v*) acetone and centrifuged at 4000× *g* for 10 min at 4 °C. Finally, the air-dried protein pellet was resuspended in 1/100 of culture volume of ice-cold 1X PBS and stored in smaller volumes until next use. Both intracellular and extracellular protein fractions were proceeded to quantification by Bradford assay protocol (Bio-rad, USA) for SDS-Page analysis.

### 4.7. Characterization of Protein Products by SDS Page and Western Blotting

For the SDS Page assay, a transfer sandwich was prepared in a Novex^®^ Semi-Dry Blotter (Invitrogen, Waltham, MA, USA) and the normalized denatured intracellular and extracellular proteins were run at 65.0 mA for 75 min. After that, the PVDF membrane containing the desired proteins was subjected to the Western blotting assay. The membrane was blocked with 2% (*w*/*v*) Bovine Serum Albumin (BSA) dissolved in 1X PBS and 0.1% Tween-20 (PBST) solution for 1 h at RT with agitation and washed. Detection of target protein on the membrane was then performed by incubation with primary antibody (1:1000 mouse anti-His·Tag^®^ Monoclonal Antibody, Novagen, Madison, WI, USA) at 4 °C overnight without agitation and washed. The membrane was incubated with a secondary antibody (1:1000 horseradish peroxidase (HRP) conjugated goat anti-mouse IgG, Amresco, Cleveland, OH, USA) for another 1 h at RT with agitation and washed. The brown-coloured chromogenic protein bands were finally developed by incubation briefly with a substrate mixture containing a tablet of 3,3’-Diaminobenzidine (DAB) (Amresco, USA) dissolved in 10 mL of PBST solution and a drop of hydrogen peroxide. The membrane was washed and an image was captured.

### 4.8. Detection of Extracellular Proteins by Enzymatic Activity Plate Assay

To qualitatively determine the secretion activity of NUC, a nuclease plate enzymatic assay was performed according to Ng and Sarkar, 2013 [10], with minor modifications. A single colony of recombinant *L. lactis* producing the nuclease enzyme and a single colony of *L. lactis* carrying the empty vector, pNZ8048, as control, were inoculated on brain heart infusion (BHI) agar (Sigma Aldrich, St. Louis, MO, USA) supplemented with Cm at 30 °C overnight. *L. lactis* overnight cultures were carefully spotted onto a new petri dish containing BHI agar supplemented with 7.5 ug/mL of Cm in triplicates. The petri dish was further incubated at 30 °C overnight. A nuclease plate enzymatic assay was then developed using the toluidine blue DNA (TBD)-BHI overlay method. A molten TBD agar (HiMedia, Mumbai, India) containing 12.56 g/L of deoxyribonuclease (DNA) substrate supplemented with 40 ng/mL nisin was prepared and was carefully overlaid without swirling on top of a BHI culture plate containing the overnight colonies of recombinant *L. lactis* and a colony of control *L. lactis*. The overlaid TBD-BHI plates were then incubated at 37 °C, and the development of a pink halo zone surrounding the recombinant *L. lactis* colonies was observed after overnight incubation. The diameter (cm) of a halo zone that was produced and surrounded a colony was measured with a ruler, and an image was captured using the Gel Doc system (Alpha Innotech, San Leandro, CA, USA).

### 4.9. Quantification of Secretion Efficiency and Yield by Fluorescence Resonance Energy Transfer (FRET) Assay

To quantitatively determine the activity of NUC, a FRET assay was conducted as described by Kiedrowski et al. (2011) [21]. First, a FRET substrate consisting of a 15-base oligonucleotide (5′- CCC CGG ATC CAC CCC-3′) with modifications at 3′-end with a Cy3 fluorophore and 5′-end with Black Hole Quencher 2 (BHQ2) was synthetically generated (1st Base Laboratories, Seri Kembangan, Selangor, Malaysia). The 15-base oligonucleotide sequence was adapted from Kiedrowski et al. (2011) [21] and served as a DNA substrate for the NUC enzyme. Meanwhile, both the Cy3 and BHQ molecules function as signal donor molecules and signal acceptor molecules, respectively. The lyophilized FRET substrate was diluted in 1X PBS and stored until the next use. To prepare the FRET mixture assay, 25 µL of 2 µM of FRET substrate diluted in 20 mM Tris (pH 8.0) and 10 mM CaCl_2_ were mixed with 25 µL of intracellular and extracellular protein extracts or 1X PBS control, respectively. The protein extracts were initially prepared from an expression of the recombinant *L. lactis* at OD_600nm_ = 0.5 with 40 ng/mL nisin for 4 h. Following centrifugation at 4000× *g*, for 10 min, at 4 °C, the culture supernatant was used directly as an extracellular protein fraction, while the cell pellet was washed, recovered in 1/10 of culture volume with 1X PBS, and lysed by sonication method before used as an intracellular protein fraction for the FRET assay. The sample mixtures were then added to the wells of a 96-well microtiter plate (Corning, Corning, NY, USA) under limited light conditions and incubated briefly for the reaction to take place. The fluorescently labelled proteins were then measured as a fluorescence unit (FU) at excitation absorbance of 552 nm and emission absorbance of 580 nm using a Tecan Infinite 200 M fluorescence plate reader (Tecan, Männedorf, Switzerland) pre-set at 30 °C. The FU unit was converted to a Unit of NUC activity per mL (U/mL) using a standard curve of fluorescence unit (FU) against known concentrations (0, 0.025, 0.25, 0.75, and 1.0 U/mL) of purified nuclease of *Staphylococcus aureus* strain ATCC #27735 (Integrated DNA Technologies, Coralville, IA, USA).

From the unit activity (U/mL) obtained in the FRET assay, secretion efficiency and total yield of NUC enzyme were measured. The total yield is reported as a sum of the unit activity of intracellular and extracellular NUC (U/mL). Secretion efficiency, SE, is calculated as shown in the equation below:

$$\mathrm{SE}\;=\;\frac{\mathrm{Unit}\;\mathrm{activity}\;\mathrm{of}\;\mathrm{extracellular}\;\mathrm{NUC}\;(U/\mathrm{mL})}{\mathrm{Total}\;\mathrm{yield}\;(\mathrm{unit}\;\mathrm{activity}\;\mathrm{of}\;\mathrm{intracellular}\;\mathrm{and}\;\mathrm{extracellular}\;\mathrm{NUC})\;\left(U/\mathrm{mL}\right)}\;\times \;100\%$$

All experimental data were reported as means of three kinetic replicates (*n* = 3) ± standard deviations. Data were subjected to statistical analysis using Student’s paired *t*-test and with a significant threshold value of *p* < 0.05.

## 5. Conclusions

Altogether, the enhancement of secretion efficiency in *L. lactis* by site-directed mutagenesis of a heterologous signal peptide has been successfully shown. It is noteworthy that there have been very few signal peptides with better ability than the most commonly used signal peptide, USP45, that have been developed in *L. lactis.* This study shows that the novel signal peptide SPK1 of *Pediococcus pentosaceus* and several of its new derivates offer an alternative to the USP45 for enhanced secretion in *L. lactis*. A better understanding of the nature of signal peptides, the nature of the mature protein, and the Sec-pathway system used in *L. lactis* have also been discovered. The role of charge balance within the tripartite domains of a signal peptide, especially the C-region, as well as in between the signal peptide and mature protein moieties surrounding the cleavage site is presumed to be the key to efficient and successful protein secretion. Additionally, in the development of new signal peptides, the biostatistical analysis tools such as SignalP is a reliable and cost-efficient tool that can be applied in predicting and understanding the functionality of the SPs before the experimental setting. For future study, the optimized *lactococcal* secretion system via the newly developed SP SPK1 variant (SPKM19) has the potential to be further explored using different heterologous enzymes or therapeutic proteins in an effort to develop GRAS *L. lactis* as an efficient cell factory for protein secretion for large-scale industrial production and oral vaccine delivery applications.

## Figures and Tables

**Table 1:** Computational analysis of signal peptides used in this study.

Signal Peptide	Aa	SPase I Cleavage Position	SPase I Cleavage Site	D-Score	pI	Net Charge SP	First 10aa Charge MP	GRAVY Index	Aliphatic Index	Instability Index
USP45 *	27	27–28	VYA-GT	0.700	10.0	+3	−1	1.174	141.11	50.14
SPK1 *	23	22–23	VHA-GT	0.781	10.0	+2	−1	1.552	165.22	16.09
SPKM20	23	23–24	VHA-GT	0.673	10.0	+2	−1	1.126	131.30	7.71
SPKM6	23	23–24	VHA-GT	0.781	10.0	+2	−1	1.704	165.22	16.09
SPKM9	26	25–26	VHA-GT	0.839	10.0	+2	−1	1.188	146.15	36.50
SPKM16	24	23–24	VHA-GT	0.840	10.0	+2	−1	1.562	162.50	15.83
SPKM17	25	25–26	AHA-GT	0.872	10.0	+2	−1	1.462	154.58	12.30
SPKM19	25	25–26	AHA-AG	0.893	10.0	+2	−1	1.462	154.58	12.30
SPKM22	26	26–27	AHA-AG	0.916	10.0	+2	−1	1.340	148.40	19.91
SPKM30	29	28–29	AHA-AG	0.917	10.6	+5	−1	0.779	132.50	18.85
SP		Amino acid sequence								
		N domain		H-domain				C-domain		
USP45 *		MKK KII SA		I LMS TVI LSA AAP				LSG VYA		
SPK1 *		MKK		ILT LVF IFV ISI LT				ATN VHA		
SPKM20		MKK		ILT LVF **G**FV IS**G** LT				ATN VHA		
SPKM6		MKK		IL**F** LVF IFV ISI LT				ATN VHA		
SPKM9		MKK		ILT LVF IFV ISI LT				ATN **PPP** VHA		
SPKM16		MKK		ILT LVF IFV ISI LT				A**A**TN VHA		
SPKM17		MKK		ILT LVF IFV ISI LT				A**A**TN **A**HA		
SPKM19		MKK		ILT LVF IFV ISI LT				ATN **A**HA**A**		
SPKM22		MKK		ILT LVF IFV ISI LT				A**P**TN **A**HA**A**		
SPKM30		MKK **KKK**		ILT LVF IFV ISI LT				A**P**TN **A**HA**A**		

SPK1 amino acid sequences were modified according to their N-, H-, and C-domain regions, respectively. The modified amino acid sequence is in bold. (*) indicates control signal peptides, SPK1, and USP45, while a total of eight designed putative SPK1 variants were selected for use in subsequent site-directed mutagenesis studies. The deposited amino acid sequence of SP-NUC was subjected to signal peptide probability and functional prediction (SignalP 4.0) using a D-score threshold set to D = 0.450, where values higher than the threshold indicate the presence of an SP. However, for the selection of SPK1 variants, a D-score threshold greater than 0.781 (D-score of SPK1) was used.

## Supplementary Materials

The supporting information can be downloaded at: https://www.mdpi.com/article/10.3390/ijms231710044/s1.
