# Supplementary material for: Optimization of Signal Peptide via Site-Directed Mutagenesis for Enhanced Secretion of Heterologous Proteins in Lactococcus lactis
Source: Int J Mol Sci. 2022 Sep 2;23(17):10044. doi: 10.3390/ijms231710044 (PMC9456127; doi:10.3390/ijms231710044)
Supplement: Supplementary file 1 [file ijms-23-10044-s001.zip › ijms-1871440-supplementary.pdf]

6.0. Supplementary Materials

Supplementary Table S1: Site-directed mutagenesis on the N-, H-, and C-regions of SPK1

Regions	Spk1 derivatives	Sequences	D-score value
N-region	Spkmut1	MKK **KKK** ILT LVF IFV ISI LT ATN VHA	0.780
N-region	Spkmut2	MK**H** ILT LVF IFV ISI LT ATN VHA	0.751
N-region	Spkmut3	MK**N** ILT LVF IFV ISI LTA TN VHA	0.747
N-region	Spkmut4	M**H**N ILT LVF IFV ISI LT ATN VHA	0.710
H-region	Spkmut5	MKK IL**L** LVF IFV I**L**I LT AT**A** VHA	0.724
H-region	Spkmut6	MKK IL**F** LVF IFV ISI LT ATN VHA	0.781
N-, H-, C-regions	Spkmut7	M**H**N IL**L** LVF IFV I**L**I LT AT**A** VHA	0.644
H-region	Spkmut8	MKK ILT LVF IFV I**L**I LT ATN VHA	0.759
H-, C-regions	Spkmut9	MKK ILT LVF IFV I**S**I LT ATN **PPP** VHA	0.839
H-, C-regions	Spkmut10	MKK IL**L** LVF **G**FV I**L**I LT AT**A** VHA	0.665
C-region	Spkmut11	MKK ILT LVF IFV ISI LT AT**A** VHA	0.707
H-, C-regions	Spkmut12	MKK ILT LVF **G**FV ISI LT AT**A** VHA	0.643
H-, C-regions	Spkmut13	MKK **G**LT LVF **G**FV ISI LT AT**A** VHA	0.627
H-, C-regions	Spkmut14	MKK IL**L** LVF IFV I**L**I LT AT**A** **A**HA	0.786
N-, H-, C-regions	Spkmut15	MKK **KKK** IL**L** LVF IFV I**L**I LT AT**A** **A**HA	0.789
C-region	Spkmut16	MKK ILT LVF IFV ISI LT A**A**TN VHA	0.840
C-region	Spkmut17	MKK ILT LVF IFV ISI LT A**A**TN **A**HA	0.759
N-, H-regions	Spkmut18	M**H**N IL**L** LVF IFV I**L**I LT ATN VHA	0.694
H-, C-region	Spkmut19	MKK IL**T** LVF IFV I**S**I LT ATN AHA**A**	0.893
H-region	Spkmut20	MKK ILT LVF **G**FV IS**G** LT ATN VHA	0.673
H-region	Spkmut21	MKK IL**M** LVF IFV I**V**I LT ATN VHA	0.777
C-region	Spkmut22	MKK ILT LVF IFV ISI LT A**P**TN AHA**A**	0.916
H-region	Spkmut23	MKK ILT LVF IFV I**L**I LT ATN VHA	0.759
H-region	Spkmut24	MKK IL**M** LVF IFV I**V**I LT ATN VHA	0.777
N-, H-regions	Spkmut25	M**H**N IL**L** LVF IFV II LT ATN VHA	0.728
H-, C-regions	Spkmut26	MKK IL**L** LVF IFV I**L**I LT AT**A** VHA	0.724
H-region	Spkmut27	MKK ILT LVF **G**FV IS**G** LT ATN VHA	0.673
Codon-biased (cb)	Spk1(cb)	MKK ILT LVF IFV ISI LT ATN VHA	0.781
N-region	Spkmut1(cb)	MKK **KKK** ILT LVF IFV ISI LT ATN VHA	0.780
N-, H, C-region	Spkmut7(cb)	M**H**N IL**L** LVF IFV I**L**I LT AT**A** VHA	0.644
C-region	Spkmut9(cb)	MKK ILT LVF IFV ISI LT ATN **PPP** VHA	0.839
H-region	Spkmut5(cb)	MKK IL**L** LVF IFV I**L**I LT AT**A** VHA	0.724
H-region	Spkmut20(cb)	MKK ILT LVF **G**FV IS**G** LT ATN VHA	0.673
N-, C-regions	Spkmut30	MKK **KKK** ILT LVF IFV ISI LTA **P**TN AHA**A**	0.917
C-region	Spk1-LEISS	MKK ILT LVF IFV ISI LT ATN VHA-**LEISSTCDA**	0.768

Supplementary Table S2: Expected band sizes of precursor NUC (preNuc) for each recombinant.

Recombinant	Expected precursor size, kDa
NZ-SPK1-NUC	22.6
NZ-USP45-NUC	22.8
NZ-SM6-NUC	22.6
NZ-SM9-NUC	22.9
NZ-SM16-NUC	22.7
NZ-SM17-NUC	22.7
NZ-SM19-NUC	22.7
NZ-SM20-NUC	22.5
NZ-SM22-NUC	22.8
NZ-SM30-NUC	23.2
NZ-SM32-NUC	23.0
NZ-SPK1-LEISS-NUC	23.6

Supplementary Table S3: List of bacterial strains and plasmids used/or developed in this study.

Bacterial strains and plasmids	Relevant features	Reference
**Strains**		
E. coli TOP10	*F- mcrA (mrr-hsdRMS-mcrBC) 80lacZ M15 lacX74 recA1 ara 139 (ara-leu)7697 galU galK rpsL (StrR) endA1 nupG*	Invitrogen, USA
L. lactis subsp. *cremoris* NZ9000	*L. lactis* subsp. *cremoris* MG1363 derivative (contains *nisRK* genes in chromosome), plasmid free	De Ruyter et al., (1996)
**Plasmids**		
pBSK(+) Simple-Amp-*Nuc*	pBluescript II SK(+) ori *E. coli* plasmid, Amp^r, DNA fragment encoding *S. aureus Nuc* gene, 3.6 kbp	Biomatik, Canada
pCR™-Blunt II-TOPO®	Kn^r, pUC ori derivative containing *lacZα-ccdB* suicide gene, 3.5kb	Invitrogen, USA
pEASY®-Blunt Zero	Kn^r, pUC ori derivative containing *lacZα-ccdB* suicide gene, 3.9kb	TransGen, China
pNZ8048	Cm^r, *L. lactis* inducible plasmid containing P$_{nisA}$ promoter, 3.3kb	Kuipers et al., (1998)
pNZ-*SPK1-Nuc*	Cm^r, pNZ8048 derivative encoding SP SPK1 fused to *NUC* gene (devoid NSP) with six-histidine (*6x-His*) tag, 4.0 kb	This study
pNZ-*USP45-Nuc*	Cm^r, encodes *USP45-Nuc* cassette, 3.9 kb	This study
pNZ-*SPKM6-Nuc*	Cm^r, encodes *SPKM6-Nuc* cassette, 3.9 kb	This study
pNZ-*SPKM9-Nuc*	Cm^r, encodes *SPKM9-Nuc* cassette, 3.9 kb	This study
pNZ-*SPKM20-Nuc*	Cm^r, encodes *SPKM20-Nuc* cassette, 3.9 kb	This study
pNZ-*SPKM16-Nuc*	Cm^r, encodes *SPKM16-Nuc* cassette, 3.9 kb	This study
pNZ-*SPKM17-Nuc*	Cm^r, encodes *SPKM17-Nuc* cassette, 3.9 kb	This study
pNZ-*SPKM19-Nuc*	Cm^r, encodes *SPKM19-Nuc* cassette, 3.9 kb	This study
pNZ-*SPKM22-Nuc*	Cm^r, encodes *SPKM22-Nuc* cassette, 3.9 kb	This study
pNZ-*SPKM30-Nuc*	Cm^r, encodes *SPKM33-Nuc* cassette, 3.9 kb	This study
pNZ-*SPK1-LEISS-Nuc*	Cm^r, encodes *SPK1-LEISSTCDA* fused to *Nuc* (devoid NSP) with *6x-His* tagged, 3.9 kb	This study
pNZ-*SPKM19-LEISS-Nuc*	Cm^r, encodes *SPKM19-LEISSTCDA-Nuc* (devoid NSP) cassettes, 3.9 kb	This study

Supplementary Table S4: List of primers used in this study.

Gene	Primers	Sequence from 5′ to 3′	Ta (°c)	Amplicon size (bp)
Nuc(devoid native SP)	F- NUC (-NSP)	GCGGGTACCATGTCACAAACAGATAACGGCGTAA	51	546
	R- NUC	GGCGAGCTCTTA**GTGGTGATGATGGTGATG**TTGACCTGAA TCAGCGTTG		
SPK1	F- SPK1	CCATGGCTATGAAAAAAATATTAAC	43	83
	R- SPK1	GGTACCAGCATGTACATTCG		
USP45	F- USP45	GGCCATGGGCATGAAAAAAAAGATTATCTCAGCTATT	46	98
	R- USP45	GGGTACCAGCGTAAACACCTGACAAC		
SPKM6	F- SPKM6	CCATGGCTATGAAAAAAATATTATTTTTG	43	83
	R- SPK1	GGTACCAGCATGTACATTCG		
SPKM9	F- SPK1	CCATGGCTATGAAAAAAATATTAAC	41	92
	R- SPKM9	GGTACCAGCATGTACTG		
SPKM16	F- SPK1	CCATGGCTATGAAAAAAATATTAAC	43	86
	R- SPKM16	GGTACCAGCATGTACATTCG		
SPKM17	F- SPK1	CCATGGCTATGAAAAAAATATTAAC	45	86
	R- SPKM17	GGTACCAGCATGAGCATTCG		
SPKM19	F- SPK1	CCATGGCTATGAAAAAAATATTAAC	45	93
	R- SPKM19	GGTACCAGCAGCATGAGCATT		
SPKM20	F- SPK1	CCATGGCTATGAAAAAAATATTAAC	44	88
	R- SPKM20	GGTACCAGCATGTACATTCG		
SPKM22	F- SPK1	CCATGGCTATGAAAAAAATATTAAC	45	89
	R- SPKM19	GGTACCAGCAGCATGAGCATT		
SPKM30	F- SPKM30	GGCCATGGCTATGAAAAAAAAAAAAAAAAAT	45	102
	R- SPKM30	CCGGTACCAGCAGCATGAGC		
SPK1-LEISS	F- SPK1	CCATGGCTATGAAAAAAATATTAAC	47	110
	R- LEISS	GGTACCTGCATCACAAGTCGACGATATTTCGAGAGCATGTA CATTC		
SPKM19-LEISS	F- SPK1	CCATGGCTATGAAAAAAATATTAAC	47	113
	R- LEISS	GGTACCTGCATCACAAGTCGACGATATTTCGAGAGCATGTA CATTC		
pNZ8048	F- pNZ8048	TATTGTCGATAACGCGAGCA	55	198
	R- pNZ8048	CGTTTCAAGCCTTGGTTTTC		
pCR™-Blunt II-TOPO®	F- M13(-20)	GTAAAACGACGGCCAGT	55	200
	R- M13 pUC (-26)	CAGGAAACAGCTATGAC		
SPK1-NUC		F-SPK1, R- NUC	55	622
USP45-NUC		F-USP45, R- NUC	54	634
SPKM6-NUC		F-SPKM6, R- NUC	51	620
SPKM9-NUC		F-SPK1, R- NUC	49	617
SPKM16-NUC		F-SPK1, R- NUC	49	623
SPKM17-NUC		F-SPK1, R- NUC	49	623
SPKM19-NUC		F-SPK1, R- NUC	49	623
SPKM20-NUC		F-SPK1, R- NUC	49	617
SPKM22-NUC		F-SPK1, R- NUC	49	623
SPKM30-NUC		F-SPKM30, R- NUC	52	632
SPK1-LEISS-NUC		F-SPK1, R- NUC	49	647
SPKM19-LEISS-NUC		F-SPK1, R- NUC	49	647

The sequences of the restriction enzyme site, *Nco*I (CCATGG), *Kpn*I (GGTACC), and *Sac*I (GAGCTC) were underlined whereas the six-Histidine tagged sequence was bolded. T_a represents optimal annealing temperature (°C).

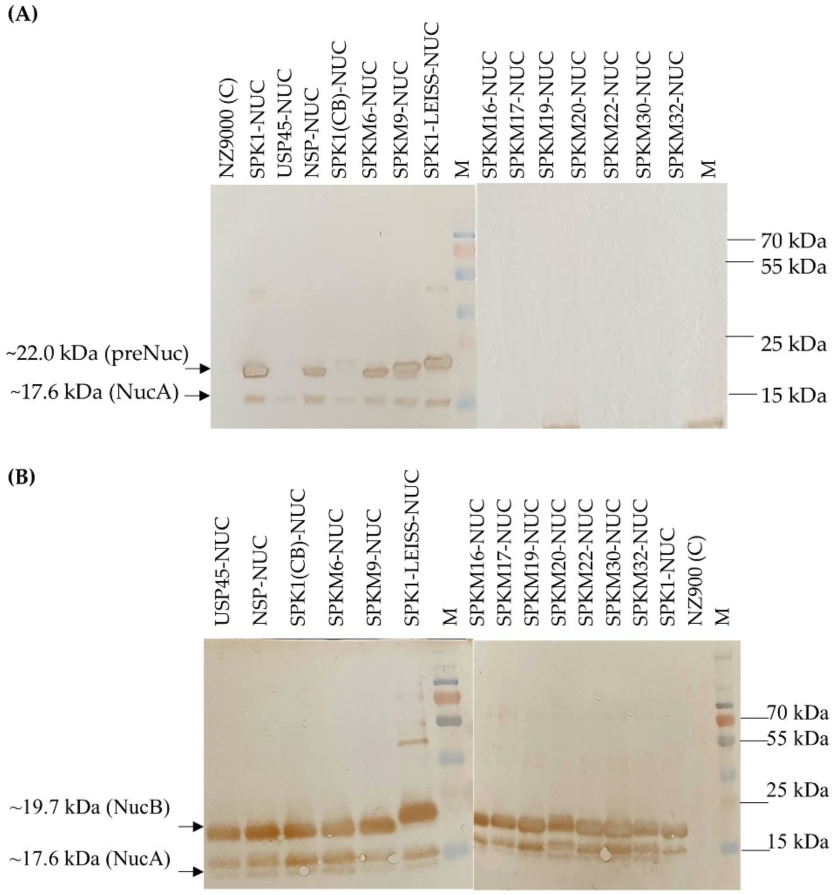

Scheme S1. Western Blotting analysis of **(A)** intracellular fraction **(B)** extracellular fraction of recombinants *L. lactis* developed in this study. Intracellular proteins fraction from cell lysate and extracellular proteins fractions from culture supernatant were determined following 4 h induction with 40 ng/ml nisin. C: Negative control (NZ9000 with empty plasmid pNZ8048); M: PageRuler™ Plus Prestained Protein Ladder. The expected precursor Nuc (preNuc) size (preNuc) for each of the constructs is listed in Supplementary Table S3, the expected size of secreted NUC (NucB) was ~19.7 kDa, while the expected size of processed NUC (NucA) was ~17.6 kDa, for all constructs. Recombinants NZ-NSP-NUC, NZ-SPK1cb-NUC, and NZ-SPKM32-NUC were not analysed in this study, thus were not further reported.

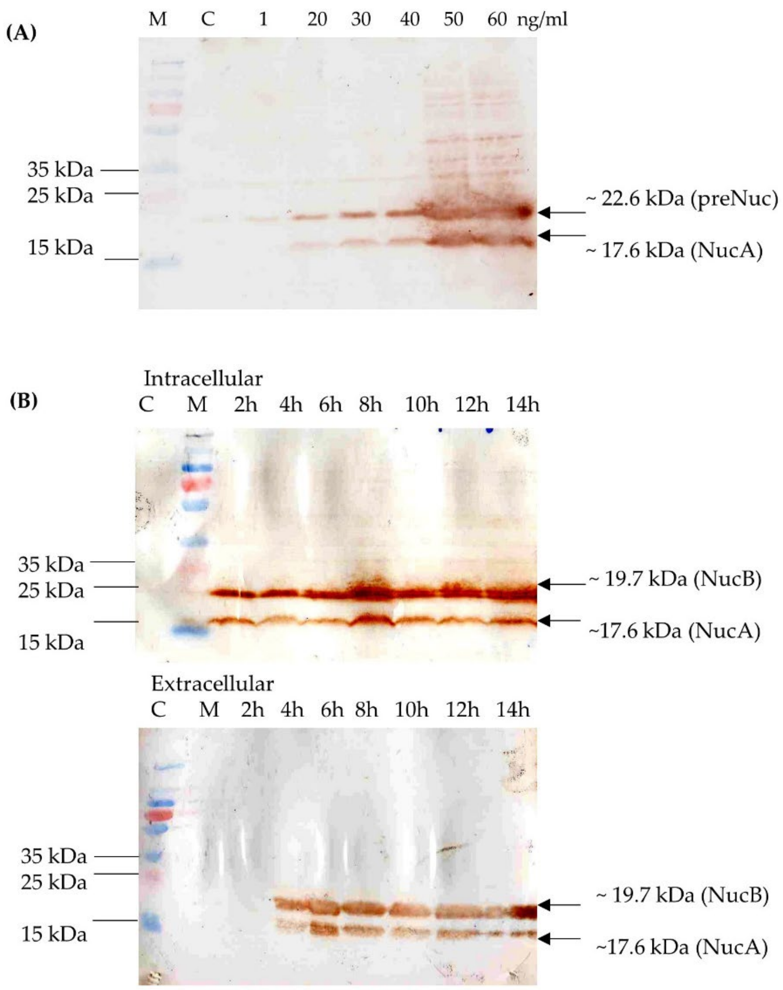

Scheme S2: Optimization of protein expression of recombinants *L. lactis* developed in this study by varying; **(A)** the nisin concentration (1, 20, 30, 40, 50 and 60 ng/ml); and **(B)** the induction time (2, 4, 6, 8, 10, 12, 14 hours). C: Negative control (uninduced NZ-SPK1-NUC); M: PageRuler™ Plus Prestained Protein Ladder. The expected size of secreted NUC (NucB) was ~19.7 kDa, while the expected size of processed NUC (NucA) was ~17.6 kDa, for all constructs. Nisin concentration 40 ng/ml was selected as the optimal concentration for production of target protein, NUC. The desired preNUC and secreted NucB were produced from 2 h and 4 h expression onwards, respectively.
